# Supplementary material for: Dissociating the Impact of Movement Time and Energy Costs on Decision-Making and Action Initiation in Humans
Source: Front Hum Neurosci. 2021 Nov 1;15:715212. doi: 10.3389/fnhum.2021.715212 (PMC8592235; doi:10.3389/fnhum.2021.715212)
Supplement: Supplementary file 1 [file Data_Sheet_1.docx]

**Supplemental Figure**

***
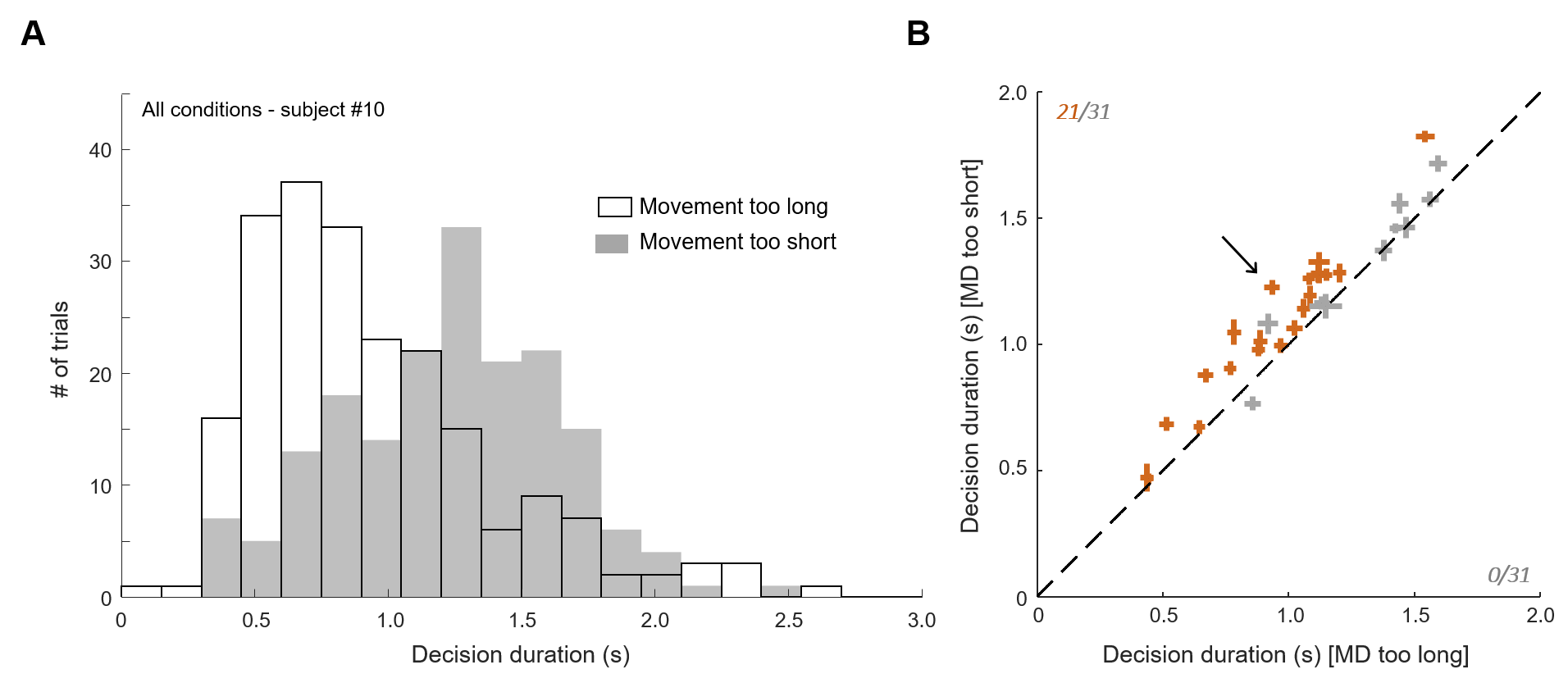
***

**Supplemental Figure 1: Decision duration in error movement trials. A.** Distribution of one example subject’s decision durations with trials sorted according to the reaching error type, i.e. too long or too short. Trials performed in the three motor conditions are included. **B.** Mean ± standard error (SE) decision duration for each subject in trials during which movements were either too long (abscissa) or too short (ordinate). The arrow indicates the subject shown in A. Same convention as in Figures 3 and 4 in the main text.

Supplemental Figure 1 shows that the too short movements were overall made when decisions were long, and the too long movements were made when decisions were short (1150 ± 59ms versus 1042 ± 57ms, |z|=12.5, p<0.001), suggesting that many subjects (21/31, p<0.05) were primarily concerned about computing a global trial duration rather than computing decision and action durations separately.
